# Use of “one-pot, mix-and-read” peptide-MHC class I tetramers and predictive algorithms to improve detection of cytotoxic T lymphocyte responses in cattle

**DOI:** 10.1186/1297-9716-45-50

**Published:** 2014-04-28

**Authors:** Nicholas Svitek, Andreas Martin Hansen, Lucilla Steinaa, Rosemary Saya, Elias Awino, Morten Nielsen, Søren Buus, Vishvanath Nene

**Affiliations:** 1 International Livestock Research Institute (ILRI), P.O. Box 30709, Nairobi 00100, Kenya; 2 Department of International Health, Immunology and Microbiology, University of Copenhagen, Copenhagen, Denmark; 3 Center for Biological Sequence Analysis, Danish Technical University, Copenhagen, Denmark; 4 Universidad Nacional de San Martín, San Martín, Buenos Aires, Argentina

## Abstract

Peptide-major histocompatibility complex (p-MHC) class I tetramer complexes have facilitated the early detection and functional characterisation of epitope specific CD8^+^ cytotoxic T lymphocytes (CTL). Here, we report on the generation of seven recombinant bovine leukocyte antigens (BoLA) and recombinant bovine β2-microglobulin from which p-MHC class I tetramers can be derived in ~48 h. We validated a set of p-MHC class I tetramers against a panel of CTL lines specific to seven epitopes on five different antigens of *Theileria parva*, a protozoan pathogen causing the lethal bovine disease East Coast fever. One of the p-MHC class I tetramers was tested in ex vivo assays and we detected *T. parva* specific CTL in peripheral blood of cattle at day 15-17 post-immunization with a live parasite vaccine. The algorithm *NetMHCpan* predicted alternative epitope sequences for some of the *T. parva* CTL epitopes. Using an ELISA assay to measure peptide-BoLA monomer formation and p-MHC class I tetramers of new specificity, we demonstrate that a predicted alternative epitope Tp2_29-37_ rather than the previously reported Tp2_27-37_ epitope is the correct Tp2 epitope presented by BoLA-6*04101. We also verified the prediction by *NetMHCpan* that the Tp5_87-95_ epitope reported as BoLA-T5 restricted can also be presented by BoLA-1*02301, a molecule similar in sequence to BoLA-T5. In addition, Tp5_87-95_ specific bovine CTL were simultaneously stained by Tp5-BoLA-1*02301 and Tp5-BoLA-T5 tetramers suggesting that one T cell receptor can bind to two different BoLA MHC class I molecules presenting the Tp5_87-95_ epitope and that these BoLA molecules fall into a single functional supertype.

## Introduction

CD8^+^ cytotoxic T lymphocytes (CTL) play a significant role in controlling intracellular pathogens such as viruses and parasites through a perforin/granule mediated cytolytic mechanism, Fas-induced apoptosis or by release of IFN-γ [1]. The specificity of CTL is mediated by their T-cell receptor (TCR) which recognize foreign peptide epitopes associated with major histocompatibility complex (MHC) class I molecules at the surface of infected cells (reviewed in [2]). The antigen specificity of CTL is commonly measured using synthetic peptide epitopes in IFN-γ ELISpot or cytolytic assays. However, the demonstration that soluble peptide-MHC (p-MHC) class I complexes will bind to TCR has led to generation of multimeric forms of p-MHC class I tetramers as diagnostic reagents that can be used to detect and purify epitope specific CTL. The importance of these reagents in research is highlighted by the creation of a NIH Tetramer Core Facility, which provides to the scientific community pre-made and made-to-order p-MHC class I tetramers for a number of human, mouse, macaque and chimpanzee MHC alleles, including class I, class II and non-classical MHC molecules. Many p-MHC class I tetramers are now also commercially available. However, such tools are in general not available for livestock species.

Peptide-MHC class I complexes are hetero-trimeric in nature consisting of the MHC class I molecule, often referred to as the heavy chain (HC), a peptide of eight to eleven amino acids in length and an invariant molecule called β2-microglobulin (β2m) (reviewed in [3,4]). Generation of the p-MHC class I complex in vivo involves many steps and is tightly regulated by chaperones [5]. Peptides are generated by the proteasome, a multi-catalytic complex in the cytoplasm that cleaves proteins into polypeptides [6], which are further processed by ER-luminal peptidases [7] during peptide loading onto MHC class I molecules by the peptide-loading-complex [8] prior to export to the cell surface. However, it is possible to generate p-MHC class I complexes at high efficiency in vitro [9-13] by co-incubation of recombinant MHC class I HC, synthetic peptide and recombinant β2m. The affinity of peptides that bind to the peptide-binding groove in the α1 − α2 domain of the HC is primarily dictated by the identity of amino acids at “anchor positions” and sequence motifs describe peptides that fit into a particular peptide-binding groove [14]. Sequence polymorphisms in this region of the HC result in different peptide binding specificities and motifs, which mediate the functional differences between different MHC class I molecules [15]. These observations have led to the generation of several algorithms ([16-18], for a detailed review see [19]) that attempt to predict the presence of CTL epitopes in proteins.

Different methods have been developed for generating multimeric peptide MHC complexes [13,20,21]. Here we describe the use of the system of Leisner et al. [11] for production of bovine leukocyte antigen (BoLA) p-MHC class I tetramers to facilitate the study of immune bovine CTL as part of our subunit vaccine development program against East Coast fever (ECF), a lethal disease of cattle in sub-Saharan Africa. The disease kills about one million animals each year with severe negative socio-economic impact on the smallholder farmers and pastoralists whose livelihoods depend on their animals [22,23]. Animals that recover from infection are solidly immune to re-infection and this observation led to development of a live parasite based infection and treatment method (ITM) of vaccination against ECF [24]. The immunity induced by ITM is primarily dependent on parasite-specific CTL and is parasite strain specific. Vaccination with mixtures of parasite broadens the spectrum of immunity [24,25] and such a vaccine, called the Muguga cocktail, which consists of three different parasite isolates, is available on a commercial basis in some countries [26]. However, there are several problems with the live vaccine as it requires a liquid nitrogen cold chain for delivery, oxy-tetracycline treatment and is expensive ($8 ~ 12/dose). Hence, the availability of a subunit vaccine with a better product profile than the current vaccine is likely to benefit programs targeting the control of ECF.

A feature of the protective CTL response generated by ITM is that it is focused on a limited number of epitopes, the identity of which is influenced by the BoLA genotype of individual animals and the strains of the parasite used for immunisation [27]. The antigen and epitope specificity of CTL induced by the Muguga isolate of *T. parva* and restricted by ten different BoLA class I molecules in cattle has been reported [28,29], and unpublished data]. Identification of these *T. parva* CTL epitopes was carried out in a two stage process, first through screening of parasite cDNA to define relevant antigens and then through screening of synthetic overlapping peptides to define epitopes within the predicted antigen sequence [29]. Although 12 CTL epitope sequences were defined they were primarily evaluated in IFN-γ ELISpot assays and most were not subjected to systematic analyses to determine minimal epitope sequences. We previously evaluated *NetMHCpan*, a pan-predictor of CTL epitopes to assess the *T. parva* epitopes. In general we found that the predictive power of the algorithm for the known CTL epitopes was high, although the method in some cases predicted the presence of alternative epitope sequences of stronger affinity [30].

Here we report on generation of p-MHC class I tetramers based on a set of seven different recombinant BoLA MHC class I molecules for detection of CTL responses to epitopes located in five different *T. parva* antigens and the use of one p-MHC class I tetramer in ex vivo assays. A critical feature of the Leisner system is that new p-MHC class I tetramers can be made on demand with a turnaround time of ~48 h, which as described herein accelerates the ability to test candidate epitopes and define new CTL specificities. Based on predictions from *NetMHCpan* and *MHCcluster* we provide experimental evidence that redefines a CTL epitope on antigen Tp2 and that an epitope on antigen Tp5 can be presented by two closely related but distinct BoLA MHC class I molecules.

## Material and methods

### Expression and purification of BoLA class I HC and β2m molecules

The expression and purification of biotinylated BoLA class I HC molecules was performed essentially as described in [11]. Briefly, truncated versions of the BoLA class I HC without their trans-membrane domain and containing a biotinylation tag (BSP) were cloned into pET28a+. The proteins were expressed in *E. coli* BL21 (DE3) co-transformed with a pASYC vector encoding the BirA enzyme, which increases the efficiency of biotinylation of the BSP tag. Expression of HC and BirA was induced by adding IPTG (1 mM) during the last 3 h of culture. Biotin (Sigma, 100 mg/L) was added to the culture prior to inducing protein expression. Large-scale production and purification were performed as previously described [31]. Briefly, this involves culture of *E. coli* BL21 (DE3) in ECPM 1 media [32] using 2-L Labfors fermenters (Infors AG). Cell lysis was performed by high-pressure cell disruption (Z PLUS, Constant Systems, Daventry, UK). Inclusion bodies were collected by centrifugation at 17 000 *g* for 10 min at 4 °C. Washed inclusion bodies were solubilized in 20 mM Tris-HCl (pH 8.0) and 8 M urea. HC molecules were purified in 8 M Urea first on Q-Sepharose (Amersham Biosciences) then on phenyl-Sepharose column (Amersham Biosciences) and high molecular complex aggregates were removed by size exclusion chromatography on Sephacryl columns. Bovine β2m molecule was purified using the same method as for human and porcine β2m [31,33,34]. Briefly, recombinant bovine Met-β2m was dissolved in 8 M urea and purified on Q-Sepharose; refolded overnight by drop-wise dilution into 25 mM Tris, 300 mM Urea, pH 8; concentrated by tangential flow ultrafiltration (Vivaflow); and buffer-exchanged into PBS by gel filtration (Superdex 200, Amersham Biosciences). Finally, the product was concentrated and frozen at −20 °C until use.

### Production of peptide-MHC class I tetramers

Peptide-MHC class I tetramers were generated as described previously [11]. Briefly, purified BoLA HC at a final concentration of 0.25 μM was incubated with the following: β2m [1.5 μM], peptide [4 μM], EDTA (1 mM), pepstatin A (1 μM), 1.10 phenantroline (100 μM), TLCK (50 μg/mL), TPCK (100 μM), PMSF (100 μM), Lutrol (0.03%), 50 mM of Tris-maleate (pH6.6), in a final volume of 500 μL. Incubation was performed at 18 °C for 48 h and then high molecular weight complexes were removed by centrifugation with an Amicon® Ultra Centrifugal Filter Units (0.5 mL/100 K) [cat no. UFC510024] at 14 000 *g* for 10 min. The flow through was recovered and the p-MHC class I complexes were tetramerized at room temperature by incubating them with 1.5 μg of phycoerythrin (PE)-streptavidin (BD Pharmingen, cat no. 554061) or allophycocyanin (APC)-streptavidin (BD Pharmingen, cat no. 554067) starting with 0.45 μg and then adding 0.21 μg every ten min over a 50 min period at room temperature and then stored at 4 °C.

### Synthetic peptides

Peptides were purchased from Mimotopes (Victoria, Australia) at a purity of 95% and re-suspended in water containing 0.1% acetic acid at a final concentration of 10 mM and diluted to working concentrations in sterile water. The sequences of *T. parva* peptide epitopes are as in Table 1. A control known peptide sequence that binds to BoLA-6*01301, YMYRVWSPL, was determined from a bank of random nonamer peptides by a binding assay as described in [34]. The sequence of the control peptide for BoLA-3*00101 is the Tp4 CTL epitope (TGASIQTTL) which is a known binder to BoLA-3*00101 [29].

**Table 1:** List of bovine immunological reagents

**Restricting BoLA class I molecule**	**CTL Lines**	***T. parva *** **antigen**	**CTL Epitope**
1) 6*01301	BB007 & BV115	Tp1	^214^VGYPKVKEEML_224_
2) 6*04101	BW02 (Clones 5 & 7)	Tp2	^27^SHEELKKLGML_37_
3) 2*01201	592	Tp2	^49^KSSHGMGKVGK_59_
4) BoLA-T2c	Bx196	Tp2	^96^FAQSLVCVL_104_
5) BoLA-T5	BV50	Tp5	^87^SKADVIAKY_95_
6) 3*00101	Bx63	Tp8	^379^CGAELNHFL_387_
7) 1*02301	495	Tp9	^67^AKFPGMKKSK_76_

The Table lists the BoLA class I molecules that were expressed in this study, the panel of CTL lines used to test p-MHC class I tetramers and their antigen and epitope specificity. The bulk CTL lines were prepared from cattle immunized with a live parasite vaccine as previously described [29]. The peptide specificity of each CTL line was confirmed by ELISpot assay (data not shown).

### ELISA assay to measure peptide-MHC class I monomer formation

Folding of p-MHC class I complexes was assessed with an ELISA assay as described in [35]. The BoLA-6*04101 molecules [0.25 μM] and bovine β2m molecule [1.5 μM] was incubated in a 96-well plate with Tp2_27-37_ and Tp2_29-37_ peptides at various peptide concentrations ranging from 0 to 40 μM. After an incubation of 48 h at 18 °C, the complexes were transferred to another 96-well plate pre-coated with streptavidin (Nunc, cat# 436014), which captures biotinylated HC, and incubated for three hours at 4 °C. After washing, the W6/32 monoclonal antibody (Santa Cruz Biotechnology, Texas, USA,, cat # SC-32235), which binds to a monomorphic epitope on β2m but only when it is incorporated in p-MHC class I complexes, was added for 1 h at 4 °C. After washing, anti-mouse IgG coupled to peroxidase (Sigma Aldrich, cat # A9917-1ML) was added to the plate followed by incubation for an hour at room temperature and further rounds of washes. Colorimetric change was performed by adding the TMB Plus2 “Ready to Use” Substrate (Kem En Tec, cat # 4395H) for 10 min at room temperature. The reaction was stopped by adding H_2_SO_4_ (0.3 M) and optical density was measured using an ELISA plate reader at 450 nm. The concentration of MHC class I and β2m molecules used in such assays is normally in the nanomolar range [35]. However, the bovine β2m molecule used in this assay contains an extra methionine at the N-terminus that diminishes binding of the W6/32 antibody to the mature p-MHC class I complex, hence higher concentrations of recombinant molecules were used in the assay. We are in the process of generating a β2m without the extra start methionine, which should allow use of recombinant molecules in the nanomolar range.

### Bovine CTL and parasite-infected cell lines

CTL and autologous *T. parva* Muguga infected lymphocyte lines (TpM) were established as previously described [29] and maintained in RPMI containing 10% fetal bovine serum [FBS] (decomplemented; Sigma), 1% penicillin/streptomycin, 1% L-Glutamine, 1% gentamycin, 0.1% beta-mercaptoethanol. CTL lines (Table 1) were propagated by stimulating them with irradiated autologous TpM at a ratio of 1:5 to 1:10 (CTL:TpM) and supplementing the media with 2 ng/mL of recombinant human interleukin (IL)-2 (Sigma) or 15% T-cell growth factor (TCGF). Cells were incubated at 37 °C in a 5% CO_2_ humidified atmosphere. Clones 5 and 7 of the BW02 CTL line were obtained by limiting dilution.

### ELISpot assays

IFNγ ELISpot assays were conducted as described in [36]. Briefly, a monoclonal anti-bovine IFNγ antibody (Serotec, Oxford, UK, cat.no. MCA1783) was incubated overnight at 4 °C on ELISpot plates (Millipore, Billerica, MA, USA) and then blocked with RPMI containing 10% heat-inactivated FBS for 2 h at 37 °C. Peptides were added at 1 μM concentration and CTL lines at 2.5 × 10^4^ cells per well. The plates were incubated at 37 °C for 20 h. Release of IFNγ was monitored with primary rabbit polyclonal anti-bovine IFNγ antibody (Sigma–Aldrich, St. Louis, MO, USA) and secondary AP-conjugated monoclonal anti-rabbit antibody (Sigma–Aldrich, St. Louis, MO, USA, cat # A2556). Development of plates was done by addition of the substrate solution Sigma Fast (BCIP/NBT, Sigma–Aldrich, St. Louis, MO, USA).

### Flow cytometry

Flow cytometry data were acquired on a BD FACSCanto™ II instrument (BD Biosciences). Compensation controls for PE, PerCP, FITC and APC were included for automatic compensation by the FACS DIVA software. 10 μL BoLA class I tetramer was used for staining cells (between 2 × 10^5^ and 5 × 10^5^ cells per sample). CD8 staining was done by incubating cells with ILRI monoclonal antibody ILA51 (IgG1) and using rat anti-mouse IgG1-PerCP (BD; cat # 340272) as the secondary antibody. Staining for perforin was done using anti-human-perforin-FITC (BD Pharmingen, San diego, CA, USA, cat# 556577) and staining for Fas-ligand was done using primary rabbit anti-Fas ligand (Santa Cruz biotechnology, cat no. SC-957) and secondary FITC-coupled anti-rabbit IgG antibody (Sigma, MO, USA, cat# F-0382). All staining were conducted in PBS-0.5% BSA except for intracellular staining with anti-perforin where cells were incubated and stained in PBS-0.1%Saponin-10% FBS. Negative control for intracellular staining was done with anti-dansyl-FITC antibody (BD Pharmingen, San diego, CA, USA, cat# 556577). The data were analyzed using the FlowJo software version 7.6.5.

### Animal experiments

Three cattle of BoLA-A18 haplotype (typed with PCR primers [forward] 5’-CCGGGATCCGAGGACT-3’ and [reverse] 5’-CTCCATCTTGCGTTTGGA-3’ specific for BoLA-6*01301) were vaccinated by ITM [24] using the *T. parva* strain Muguga 3308. Whole blood was collected from these cattle at the day of vaccination (day 0) and every two days till appearance of tetramer positive cells. PBMC were purified on Ficoll paque density gradients at 2500 rpm for 25 min and 5 × 10^5^ PBMC were stained with the Tp1_214-224_-BoLA-6*01301 tetramer as described in the flow cytometry procedure. This animal experiment was approved by the ILRI IACUC committee [# 2012.10].

### NetMHCpan and MHCcluster

We used *NetMHCpan*[37], a pan-specific predictor trained on known peptide epitope and MHC class I sequence data and the affinity of p-MHC class I interactions to predict *T. parva* epitopes. The binding affinity of selected epitopes was predicted using the latest version of *NetMHCpan* (version 2.8, [38]). Predictions of strong peptide binders was done by evaluating all possible eight to eleven-mers from antigens Tp2 and Tp5 with BoLA-6*04101, and BoLA-T5 and BoLA-1*02301 respectively. We also used *MHCcluster*[39] to compare predicted peptide-binding specificities between the different BoLA class I molecules involved in this study.

### Comparison of sequence similarities between BoLA-T5 and BoLA-1*02301

BoLA-1*02301 and BoLA-T5 amino acid sequences were downloaded from the Immuno-Polymorphism Database [40-42] and NCBI respectively, and a MUSCLE alignment was done using clcSequence Viewer 6.0. Visualization of the position of the amino acid differences between these molecules was done on the crystal structure of BoLA-2*01801 [43] using RasWin.

## Results

### Generation of peptide-MHC class I tetramers and assay on defined CTL lines

The “on-demand” Leisner system for tetramer generation involves p-MHC class I monomer formation in the presence of excess peptide (concentration of 4 μM), followed by tetramerization using either PE or APC labelled streptavidin (see Materials and methods). This method was used to generate a set of seven p-MHC class I tetramers from recombinant BoLA class I HC and bovine β2m and synthetic peptides as listed in Table 1. This system does not routinely measure monomer formation but this became necessary for some experiments (see below).

**Figure 1:**
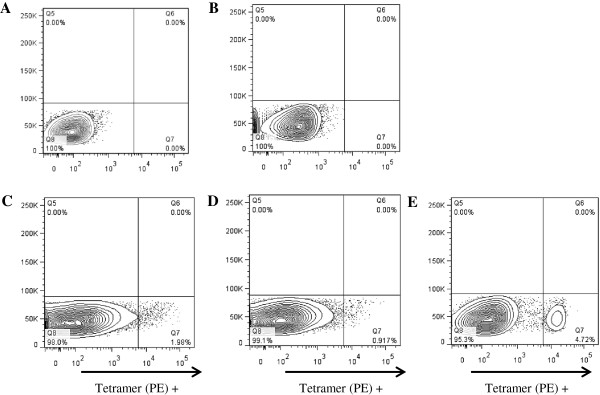
**Tp1**_**214-224**_-BoLA-6*01301 p-MHC class I tetramer staining of bovine CTL line BB007. The CTL line was stained with buffer alone (panel **A**), p-MHC class I tetramers made with the control peptide, YMYRVWSPL (panel **B**), p-MHC class I tetramers made with the peptide Tp2_96-104_, which is restricted by another BoLA molecule [BoLA-T2c] (panel **C**), p-MHC class I tetramers made in the absence of peptide (panel **D**) and p-MHC class I tetramers made with peptide Tp1_214-224_ (panel **E**). All tetramers were generated with PE-streptavidin.

To verify the specificity of p-MHC class I tetramers we used *T. parva*-epitope-specific bulk CTL lines generated from ECF immune cattle as described in former studies [28,29,44]. To confirm the epitope specificity of the CTL lines, an IFN-γ ELISpot assay was performed using the peptides described in Table 1. As expected all CTL lines were positive in this assay but only in the presence of their respective peptide epitope (data not shown). We then tested the specificity of the novel BoLA p-MHC class I tetramers to the different bovine CTL lines. First, we used the Tp1_214-224_-BoLA-6*01301 p-MHC class I tetramer and BB007 CTL line to control for proper staining (Figure 1). As negative controls we used unfolded tetramerized HC (absence of peptides or an irrelevant peptide, Tp2_96-104_, that does not bind BoLA-6*01301) and tetramerized HC folded with a control peptide, YMYRVWSPL, known to bind to BoLA-6*01301. The control peptide was derived from a bank of random nonamer peptides that bound with high affinity to BoLA-6*01301 (affinity equal to, or greater than, 1 nM). As shown, tetramers prepared with the control peptide (Figure 1B) displayed very low staining on the CTL when compared to the non-stained sample (Figure 1A). However, when CTL were stained with tetramers made without peptide or the irrelevant peptide we observed a low level smear staining of CTL (Figure 1C and D respectively), most likely due to non-specific binding by unfolded HC. When the CTL were stained with a tetramer formed with the Tp1 peptide epitope we saw staining of a clear distinct cell population (Figure 1E). Figures 1B, C and D suggest that non-specific staining of CTL is only observed in the absence of peptides that bind to HC and that minimal if any unfolded HC remain when binding peptide is in excess. Hence, the result in Figure 1E is significant.

**Figure 2:**
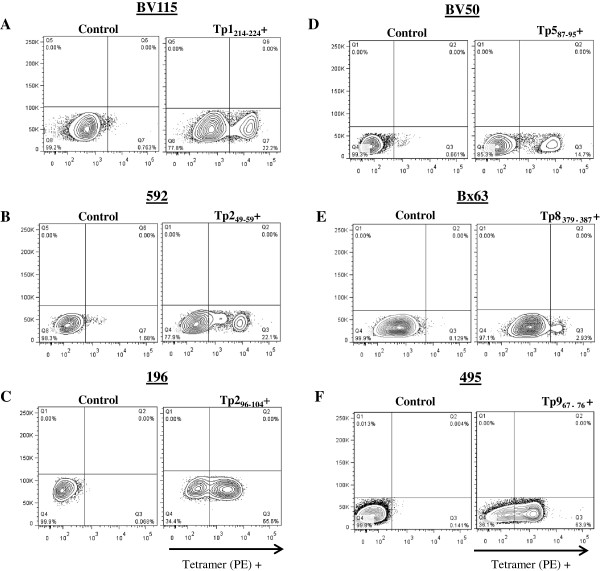
**Assay of the panel of p-MHC class I tetramers on different antigen specific CTL lines.** In each panel the left graph represents unstained negative control cells and the right graph p-MHC class I tetramer stained cells. Panel **A**: Tp1_214-224_-BoLA-6*01301 tetramers on cell line BV115; Panel **B**: Tp2_49-59_-BoLA-2*01201tetramers on cell line 592; Panel **C**: Tp2_96-104_-BoLA-T2c tetramers on cell line Bx196; Panel **D**: Tp5_87-95_-BoLA-T5 tetramers on cell line BV50; Panel **E**: Tp8_379-387_-BoLA-3*00101 tetramers on cell line Bx63; Panel **F**: Tp9_67-76_-BoLA-1*02301 tetramers on cell line 495. All tetramers were generated with PE-streptavidin.

**Figure 3:**
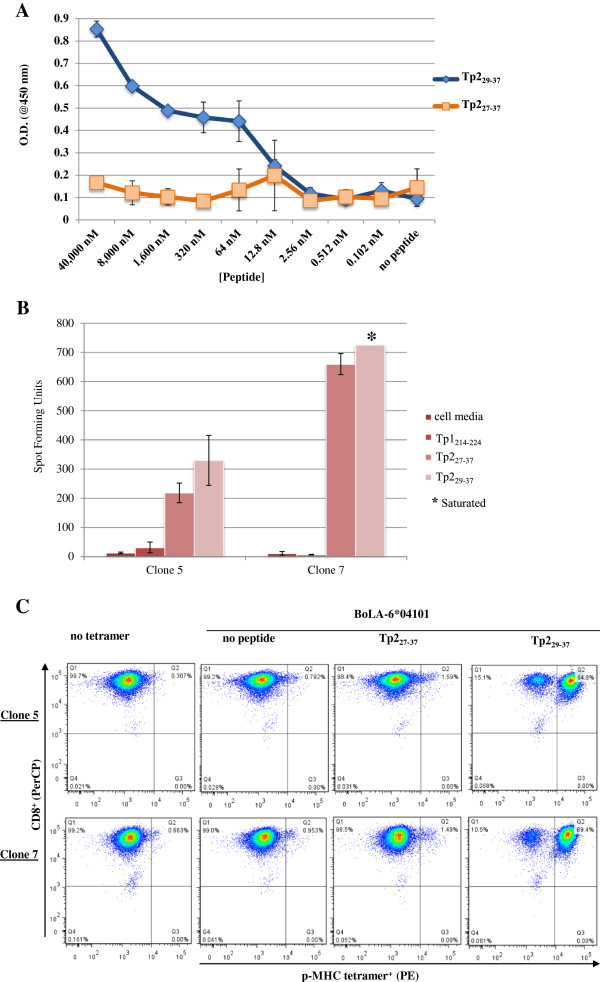
Definition of an alternative Tp2 epitope that binds to BoLA-6*04101. Panel **A**: p-MHC class I monomer formation ELISA assay using Tp2_27-37_ and Tp2_29-37_ peptides. Panel **B**: ELISpot performed on two clones (5 and 7) of the cell line BW02 with cell media alone, Tp1_214-224_, Tp2_27-37_ or Tp2_29-37_ peptides. Data are presented as spot forming units (SFU) and each well contained 2.5 × 10^4^ CTLs. Asterisk (*) indicates saturated wells. Panel **C**: Two clones of the cell line BW02 was stained with anti-bovine CD8 (PerCP) and different BoLA-6*04101 tetramers. The unstained control or stained with the unfolded (no peptide) BoLA-6*04101 tetramer are depicted on the left. Tetramer labelling was measured on the CD8-gated cell population.

Each of the remaining p-MHC class I tetramers was then tested against the CTL lines, including testing Tp1_214-224_-BoLA-6*01301 p-MHC class I tetramers on a different CTL line, BV115. Distinct tetramer-positive populations were seen in cognate lines for 6 of the 7 p-MHC class I tetramers analysed, however, with great differences in the frequency of tetramer positive cells between the different cell lines. For example, about 3% of cell line Bx63 stained positive with Tp8_379-387_-BoLA-3*00101 tetramers, while about 65% of cell line 196 stained positive with Tp2_96-104_-BoLA-T2c tetramers (Figure 2). Non-specific staining was monitored using unfolded tetramers to differentiate with specific staining (data not shown). In some cases, p-MHC class I tetramer folded with control peptides known to bind BoLA-6*01301 and BoLA-3*00101 were used as further controls. The p-MHC class I tetramers generated with BoLA-T5, BoLA-T2c and BoLA-1*02301 were controlled using an irrelevant CTL line to verify that no nonspecific staining was occurring (data not shown) since no control peptides for these BoLA molecules were available. Tp2_27-37_-BoLA-6*04101 p-MHC class I tetramers did not bind a discrete population of cells in cell line BW02 and resembled a non-specific staining profile (data not shown). We provide strong evidence below that this is likely due to Tp2_29-37_ rather than Tp2_27-37_ being the epitope that binds to BoLA-6*04101 (Figure 3).

### A role for NetMHCpan in *T. parva* CTL epitope identification

We have previously reported that the *NetMHCpan* algorithm [45] predicted an alternative epitope sequence for six of the 12 epitopes [30]. Since that time *NetMHCpan* has been improved and version 2.8 predicts alternative epitopes for only 4 of the 12 epitopes: Tp7_207-214_ instead of Tp7_206-214_, Tp2_41-48_ instead of Tp2_40-48_, Tp2_138-146_ instead of Tp2_138-147_ and Tp2_29-37_ instead of Tp2_27-37_. As a measure of predictive accuracy, we use the false positive fraction score. For a given CTL epitope, this score is defined as the number of 8-11mer peptides with a predicted binding affinity stronger than the epitope divided with the total number of 8-11mer peptides found within the source protein sequence. A false positive fraction score closer to 0 is indicative of a high predictive performance.

*NetMHCpan* predicted that Tp2_29-37_ (EELKKLGML) has a false positive fraction score of zero for BoLA-6*04101 while that for the peptide Tp2_27-37_ (SHEELKKLGML) we have been working with (Table 1) was 0.039 (this value has changed from previous predictions with older version of *Net*MHC*pan* but is still indicative of a low binding affinity [30]). To experimentally verify this prediction we developed a semi-quantitative ELISA assay as described in [35] to measure p-MHC class I monomer formation (see Materials and Methods). The W6/32 monoclonal antibody used in the ELISA assay binds to β2m from different species but only when β2m is in a complex with HC and peptide. If a stable p-MHC class I complex is not formed, for example in the absence of peptides, β2m remains free in solution and will not bind to the streptavidin-bound HC on the plate. Figure 3A clearly demonstrates that Tp2_27-37_ does not bind to BoLA-6*04101, whereas Tp2_29-37_ demonstrates a good binding curve.

We then generated p-MHC class I tetramers made with each one of the two Tp2 sequences to stain BW02 CTL line clones that in ELISpot assay respond to both peptide sequences (Figure 3B). Figure 3C shows a clear difference in staining profile between the p-MHC class I tetramer generated with Tp2_29-37_ and the p-MHC class I tetramer generated with Tp2_27-37_, with the latter showing a profile similar to that seen for non-specific staining of cells. Thus, our results strongly support the *NetMHCpan* prediction that Tp2_29-37_ and not Tp2_27-37_ is the correct epitope sequence for BoLA-6*04101.

### Tp1-tetramer positive CTL are also positive for CD8, FasL, and perforin

**Figure 4:**
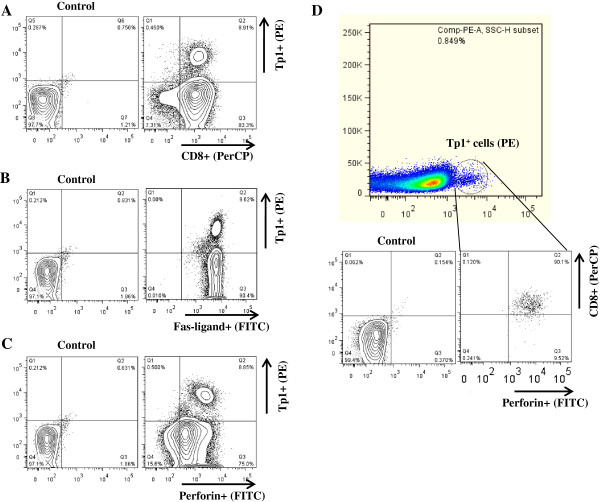
**Multiple staining of Tp1**_**214-224 **_**peptide-MHC tetramer positive cells.** In each panel the left graph represents unstained negative control cells. The cell line BB007 was stained with Tp1_214-224_-BoLA-6*01301 tetramer in conjunction with anti-bovine CD8 (panel **A**), anti-Fas-ligand (panel **B**), or anti-perforin (panel **C**). Panel **D**: BB007 CTL line stained with Tp1_214-224_-BoLA-6*01301 tetramer and with anti-bovine CD8 and anti-perforin. Data in Panels **A**, **B** and **C**, and data in Panel **D** are from two independent experiments.

It has been shown that active CTL express both perforin and Fas-ligand [1,46,47]. In order to determine if the cells identified with the tetramers were of the CD8 lineage and expressed cytotoxicity markers, we performed multiple staining of cells. For example, all Tp1 positive cells in the cell line BB007 were CD8-positive (Figure 4A), FasL-positive (Figure 4B) or perforin-positive (Figure 4C). A triple staining confirmed that tetramer-positive cells are also positive for CD8 and perforin (Figure 4D). For the 592, Bx63 and BV50 CTL cell lines analysed, all tetramer positive cells were also positive for CD8 but other markers were not tested (data not shown). We have demonstrated a correlation between the presence of perforin in tetramer-positive cells and specific cytotoxic activity in BV115 and BB007 cell lines as well as from cells isolated ex vivo (data not shown, manuscript in preparation).

### Ex vivo analysis of CTL following ITM vaccination of cattle

**Figure 5:**
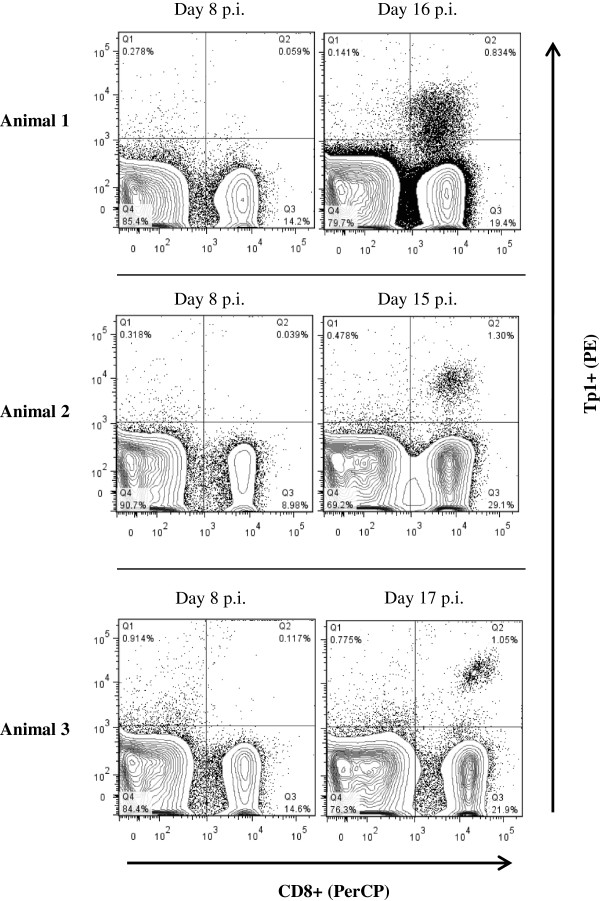
**Identification of Tp1**_**214-224 **_**peptide-MHC tetramer positive cells in ex vivo assays.** PBMC were isolated from three BoLA-A18 cattle vaccinated with the live *T. parva* (Muguga 3308 strain) and stained with Tp1_214-224_-BoLA-6*01301 tetramers at day 8 and day 15-17 post infection (pi). Tetramer labelling was measured on the CD8-gated population.

With the validation of p-MHC class I tetramers, we wanted to determine if we could detect antigen specific CTL directly isolated from cattle. Three BoLA-A18 positive animals that were predicted to mount a CTL response to antigen Tp1 were vaccinated by the ITM method [reviewed in [24]] using *T. parva* strain Muguga 3308. Appearance of Tp1 specific CTL in peripheral blood was monitored every two days following infection using Tp1_214-224_-BoLA-6*01301 tetramers (Figure 5). As illustrated, Tp1 positive cells were successfully identified on days 15 to 17 at a frequency of about 1% of PBMC. The appearance of tetramer-positive cells correlated with previous reports of cytotoxic activity towards *T. parva* peaking at day 18 post-infection [48-50].

### Predicted peptide binding specificity as a means of clustering of the seven BoLA MHC class I molecules

**Figure 6:**
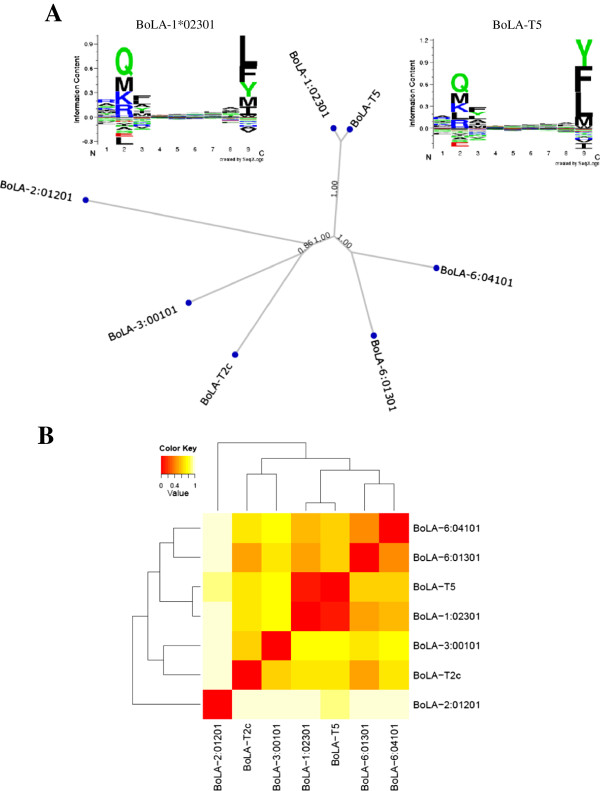
*MHCcluster *prediction of the functional similarity of BoLA-T5 and BoLA-1*02301. Panel **A**: UPGMA tree-based clustering of the relationship between the seven BoLA molecules (sequence logo representations of the predicted binding motifs of BoLA-T5 and BoLA-1*02301 were generated by *MHCcluster* using Seq2Logo [52]); Panel **B**; heat-map visualization of the degree of functional relationship between the seven BoLA molecules, red colour shows closer functional relationship.

We used the *MHCcluster* 2.0 server [51] to determine the functional relationship of the different BoLA class I molecules based on their predicted peptide binding specificities. According to this analysis BoLA-T5 and BoLA-1*02301 cluster together as depicted in the dendrogram (Figure 6A). Their similarity in function is more apparent in the sequence logo of the amino acid motifs predicted to bind the two BoLA molecules (Figure 6A) and the heat-map representation of the degree of functional relationship between the different BoLA molecules (Figure 6B).

**Figure 7:**
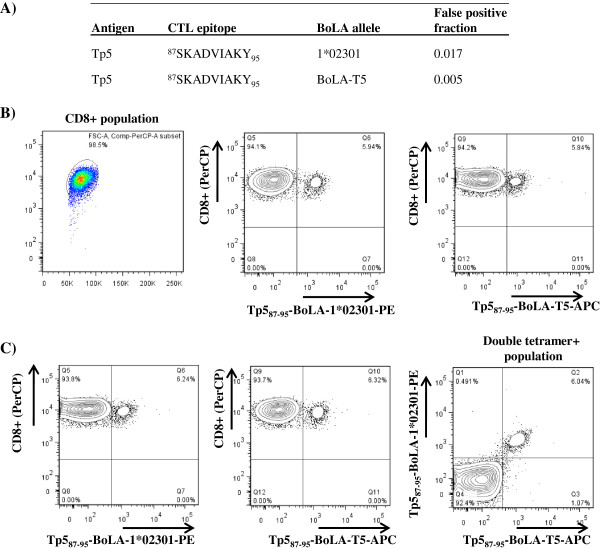
**The Tp5**_**87-95 **_epitope is presented by two BoLA molecules (BoLA-T5 and BoLA-1*02301). Panel **A**: predicted binding affinity of Tp5_87-95_ peptide epitope for BoLA-1*02301 and BoLA-T5; Panel **B**: The cell line BV50 stained with anti-bovine CD8 (PerCP) and Tp5_87-95_ p-MHC class I tetramers made with BoLA-1*02301 or BoLA-T5. Panel **C**: The cell line BV50 co-stained with anti-bovine CD8 (PerCP), Tp5_87-95_-BoLA-1*02301 tetramers (PE) and Tp5_87-95_-BoLA-T5 tetramers (APC). Tetramer labelling was measured on the CD8-gated population.

**Figure 8:**
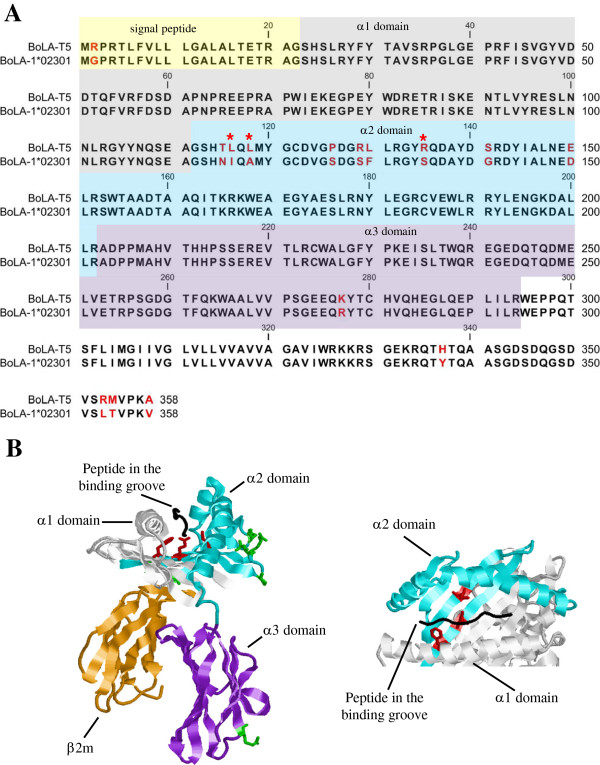
Sequence alignment of BoLA-T5 and BoLA-1*02301 molecules and identification of amino acid differences in the peptide-binding groove. Panel **A**: MUSCLE alignment of BoLA-T5 and BoLA-1*02301 amino acid sequence; differences in amino acid sequence is highlighted by the red letters; asterisks (*) in red indicate amino acids that map to the pseudo-sequence of 34 amino acids that were defined as in contact with peptide; sequence highlighted in yellow indicate the signal peptide; sequence highlighted in grey indicate the α1 domain; sequence highlighted in blue indicate the α2 domain; sequence highlighted in purple indicate the α3 domain. Panel **B**: Ribbon drawing of the BoLA-2*01801 [43] molecule with position of all amino acid differences in the α2 (highlighted in blue) and α3 (highlighted in purple) domains between BoLA-T5 and BoLA-1*02301. Amino acids that map to the pseudo-sequence are illustrated in red; amino acids that do not map to the pseudo-sequence are illustrated in green. The β2m molecule is illustrated in orange and the peptide in black.

The algorithms predicted that the CTL epitope from Tp5 [^87^SKADVIAKY_95_], which was found to be restricted by BoLA-T5 (Table 1) should also bind BoLA-1*02301 (Figure 7A). This was confirmed by p-MHC monomer binding assays (data not shown). We then generated Tp5_87-95_-BoLA-1*02301 p-MHC class I tetramers and stained BV50 and 495 CTL lines, which are restricted by BoLA-T5 and BoLA-1*02301, respectively (Table 1). The only CTL line stained by this tetramer was BV50, which is also stained by Tp5_87-95_-BoLA-T5 p-MHC class I tetramers (Figure 7B). Interestingly, both tetramers bind the same population of CTL as demonstrated by co-staining of the BV50 cell line with p-MHC class I tetramers using Tp5_87-95_-BoLA-1*02301 labelled with PE and Tp5_87-95_-BoLA-T5 labelled with APC. (Figure 7C). A MUSCLE alignment of the BoLA class I molecules revealed that there are only 15 amino acid differences between BoLA-1*02301 and BoLA-T5 (highlighted in Figure 8A by the amino acid letters in red). The close sequence identity between these two BoLA class I molecules may explain their similar peptide binding specificity (Figure 8A). However, three of the 15 amino acid polymorphisms map to the peptide binding groove in the α2 domain (Figure 8B). Nevertheless, our results establish that the Tp5_87-95_ epitope can be presented not only by BoLA-T5 but by BoLA-1*02301 as well.

## Discussion

Peptide-MHC class I tetramer complexes have become essential tools in cellular immunology to decipher and characterize epitope-specific CD8^+^ T cell immune responses [12,13,21]. They are especially useful for detection of CTL that occur at low frequency and are finding many applications in ex vivo assays for direct assessment of T cell responses. Such reagents have become indispensable tools in murine and human immunology but are generally lacking for many important livestock species. In an effort to provide reagents to fill this gap, we recently described initial efforts to develop p-MHC class I tetramers for use in swine and bovine research [30,34] using a simple in-house system that permits generation of p-MHC class I tetramers with a turnaround time of 48 h [11]. Here, we report on p-MHC class I tetramers made from seven BoLA MHC class I genes and the power of integrating the on demand tetramer system with CTL epitope prediction data from *NetMHCpan*, a pan specific algorithm, and *MHCcluster*, a method for clustering of MHC molecules based on function, i.e., their predicted peptide binding specificities, rather than sequence.

We used the novel immunological reagents we generated to facilitate our research on ECF, a lethal disease of cattle. Parasite-specific CTL induced by the ITM vaccine play a major role in mediating immunity to ECF [25,28,29]. A number of *T. parva* antigens that are the targets of CTL induced by ITM have been identified using high-throughput IFN-γ ELISpot immunoscreens of antigen presenting cells transfected with either candidate *T. parva* genes or pools of parasite cDNA libraries [29]. Positive genes were then tested in cytolytic assays and CTL epitopes mapped by use of synthetic overlapping peptides. Based on such data a panel of 12 experimentally verified epitope sequences derived from seven *T. parva* antigens and restricted by ten BoLA class I molecules have been reported [29] and unpublished data]. However, these peptide sequences were not subjected to systematic analyses to determine minimal epitope sequence data and due to a lack of appropriate reagents the epitopes were not verified in independent assays, e.g., in peptide-BoLA binding assays. The prediction of alternative sequences for some of the CTL epitopes by *NetMHCpan*[30] has raised questions regarding the performance of the algorithm as well as the veracity of CTL epitope sequences. We have addressed some of these issues.

The method of Leisner et al. works well for production of seven recombinant BoLA MHC class I molecules, adding to the list of mammalian MHC molecules generated by this system. We have made and validated the use of p-MHC class I tetramers that allow us to rapidly identify *T. parva* specific CTL responses directed to one of seven epitopes on five different antigens (Figures 1, 2, and 3). We used these reagents to demonstrate that all Tp1-specific CTL co-express CD8, FasL or perforin (Figure 4). Such assays facilitate rapid typing of the predicted functionality of *T. parva* specific CTL. This is important for subunit vaccine development studies since in the past we have demonstrated priming of peptide specific IFN-γ ELISpot CD8^+^ T cell responses in the absence of cytolytic activity [28], and only the latter activity correlates with immunity to ECF. The ability to detect cytolytic activity in peripheral blood post-vaccination is the range of 14 to 21 days [48-50]. However, several rounds of CTL in vitro stimulation are required for detection of cytolytic activity. Using the Tp1-specific tetramer we were able to detect CTL in BoLA-A18 cattle as early as day 13 post-vaccination by the live parasite based ITM vaccine (data not shown). The CTL response peaked at about day 15 (Figure 5). Thus, the Tp1-specific tetramers demonstrate high sensitivity and great potential as diagnostic tools for detecting ECF immunity in cattle with the ability to stain cells isolated ex vivo from infected animals without further stimulation and expansion in vitro. While the remaining p-MHC class I tetramers remain to be validated in ex vivo assays, we expect that our collection of p-MHC class I tetramers will permit studies on the kinetics and functionality of antigen specific CTL responses to *T. parva* during infection and facilitate immunogenicity studies during subunit vaccine trials. Moreover, as new CTL epitopes restricted by these BoLA molecules are identified, in future studies we will be able to rapidly expand the repertoire of p-MHC class I tetramers available for ECF research.

Several unexpected results were predicted through the use of *NetMHCpan* and *MHCcluster*. The first is that *NetMHCpan* predicted that Tp2_29-37_ rather than Tp2_27-37_ is likely to be the correct peptide sequence that binds to BoLA-6*04101 (Figure 3). The longer Tp2 peptide sequence works in both ELISpot and cytolytic assays (our data and [29]) but this peptide does not bind to BoLA-6*04101 (Figure 3). We presume that the peptide is processed to the functional epitope during the ELISpot assays, which does not occur during the peptide MHC binding assays or formation of p-MHC class I tetramers. *NetMHCpan* also predicted three alternative epitopes from those we have previously described: Tp7_207-214_ instead of Tp7_206-214_, Tp2_41-48_ instead of Tp2_40-48_, and Tp2_138-146_ instead of Tp2_138-147_. Due to a lack of CTL lines of appropriate specificity, we have not been able to test these predictions. However, Tp7_207-214_ has been previously shown to be negative in ELISpot assays [29]. Preliminary data from our group (not shown) has determined a higher binding affinity of Tp7_207-214_ for BoLA-T7 (Kd value of 142 nM) compared to the binding affinity of Tp7_206-214_ (Kd value of 252 nM). This discrepancy and the alternative epitopes predicted for Tp2 remains to be resolved.

One of the outputs of the *MHCcluster* server is a tree-based (Figure 6A) and heat-map (Figure 6B) visualization of the functional relationship between MHC molecules based on their predicted peptide binding specificity rather than sequence alignment of the MHC molecules themselves. For example, it is known that HLA-A*0201 and HLA A*0301 share 93% sequence identity but exhibit completely different peptide binding specificities [19]. Thus, clustering on function rather than sequence of MHC molecules is a more important parameter in CTL biology and immunology. *NetMHCpan* predicted that the epitope Tp5_87-95_ should bind to BoLA-1*02301 (with a false positive fraction of 0.017) in addition to BoLA-T5, the original molecule that was defined to present the Tp5_87-95_ epitope by parasite infected cells (Table 1). Using both peptide-BoLA binding assays and p-MHC class I tetramers we demonstrate that the Tp5_87-95_ epitope does indeed bind both BoLA molecules and that a single TCR can recognize the Tp5_87-95_ epitope presented by either BoLA molecule (Figure 7). This suggests that as for HLA molecules, it will be possible to categorize BoLA molecules into supertypes based on function. More importantly the functional overlap of BoLA molecules suggest that it should be possible to select a repertoire of *T. parva* antigens to cover a broader population of cattle than indicted by the limited specificity of CTL responses generated by the live ITM vaccine.

The finding that the Tp5_87-95_ epitope binds to two BoLA molecules was unexpected. What is more surprising was the observation that three of 15 amino acid sequence polymorphisms between the BoLA molecules map to the peptide binding groove (Figure 8B). More specifically they map to the pseudo-sequence of 34 amino acids in HLA molecules, which form the basis of the pan prediction capacity of *NetMHCpan*[37]. These 34 polymorphic residues have been defined from crystal structure studies as being within 4.0 Angstrom (Å) of the peptide in any of a representative set of HLA-A and HLA-B structures binding a nonameric peptide [37] and hence are predicted to play a critical role in peptide binding. Leucine in BoLA-T5 is replaced by isoleucine in BoLA-1*02301 at position 94; leucine by alanine at position 96 and arginine by serine at position 113 in the mature protein (highlighted with red asterisk in Figure 8A). Of these substitutions two are conservative in nature. Our data indicates that these changes and the non-conservative amino acid substitution do not have a dramatic influence on the specificity of peptide binding. The *NetMHCpan* method has, in several benchmark studies, proven to accurately predict the peptide binding motif for molecules characterised by limited or no experimental peptide binding data [16,53,54]. In addition, *NetMHCpan* can differentiate between critical and less critical amino acid substitutions in the MHC pseudo-sequence in situations where the amino acid variations have been observed and used in training the method [39]. As the training data for *NetMHCpan* increases it should become possible to predict such subtleties in peptide binding to MHC molecules.

An essential feature of the system we have described is that p-MHC class I tetramers are generated over a 48 h time frame using an easy protocol starting from the raw ingredients of recombinant MHC class I HC, synthetic peptide and recombinant β2m. We have also used the BoLA molecules to develop a simple ELISA based method to determine peptide-MHC complex formation. The power of combining such experimental verifications with predictions from *NetMHCpan* is highlighted by our results and have important implications for research on infectious diseases affecting not only cattle but other animal species including humans. We have continued to improve *NetMHCpan* using peptide-BoLA binding data (manuscript in preparation) and expect similar improvements to occur through incorporation of data from other MHC projects. These new bovine MHC class I tetramers are sensitive tools that can be used to study the immune responses in cattle e.g. the CD8^+^ T cells that play a role in *Theileria parva* immune responses or in other parasitic or viral diseases where CTL contribute to immunity [43,55]. More BoLA class I alleles could be included in the pipeline as more information about MHC class I expression in cattle of eastern, central and southern Africa is generated.

## Abbreviations

Å: Angstrom; AP: Alkaline phosphatase; APC: Allophycocyanin; β2m: Beta-2-microglobulin; BoLA: Bovine leukocyte antigen; BREAD: Basic research to enable agricultural development; BSA: Bovine serum albumin; CTL: Cytotoxic T lymphocytes; E. coli: *Escherichia coli*; ECF: East Coast fever; EDTA: Ethylenediaminetetraacetic acid; ELISA: Enzyme linked immunosorbent assay; ER: Endoplasmic reticulum; FBS: Fetal bovine serum; FITC: Fluorescein isothiocyanate; HC: Heavy chain; IFN: Interferon; IgG: Immunoglobulin-G; IL: Interleukin; IPTG: Isopropyl β-D-1-thiogalactopyranoside; ITM: Infection and treatment method; MHC: Major histocompatibility complex; NSF: National Science foundation; O.D.: Optical density; PBMC: Peripheral blood mononuclear cells; PBS: Phosphate buffered saline; PE: Phycorerythrin; PerCP: Peridinin chlorophyll protein; pi: post-infection; PLC: Peptide-loading-complex; PMSF: Phenylmethylsulfonyl fluoride; T. parva: *Theileria parva*; TCGF: T-cell growth factor; TCR: T-cell receptor; TLCK: Tosyllysine chloromethylketone; TPCK: Tosyl phenylalanyl chloromethyl ketone; TpM: *T. parva* Muguga transformed lymphocytes; UF: Unfolded.

## Competing interests

The authors declare that they have no competing interests.

## Authors’ contributions

NS and VN conceived the experiments and wrote the manuscript. NS typed the animals; prepared the tetramers; performed flow cytometry, ELISpot and binding assays; and analysed the data. AMH carried out Tp2_27-37_/BoLA-6*04101 and Tp5/BoLA-1*02301 binding assays. LS planned and supervised the animal experiment. RS and EA prepared/stimulated the CTL lines in vitro and performed ELISpot assays. MN developed *NetMHCpan* and performed predictions of peptide binding on the BoLA class I molecules. SB expressed the recombinant BoLA class I molecules and developed the “on-demand” generation of MHC-tetramer technology. All authors read and approved the final manuscript.
